# Systemic Inflammatory Biomarkers and Unsupervised Phenotyping of Surgical Pleurodesis Outcomes in Malignant Pleural Effusion: A Retrospective Single-Centre Study

**DOI:** 10.3390/diagnostics16213492

**Published:** 2026-10-25

**Authors:** Luca Frasca, Ilaria Suriano, Antonio Sarubbi, Francesco Branda, Filippo Longo, Massimo Ciccozzi, Pierfilippo Crucitti

**Affiliations:** 1 Department of Thoracic Surgery, Fondazione Policlinico Universitario Campus Bio-Medico, Via Alvaro del Portillo, 200, 00128 Rome, Italy; 2 PhD Programme in Microbiology, Immunology, Infectious Diseases, and Transplants (MIMIT), Faculty of Medicine and Surgery, University of Rome Tor Vergata, Viale Oxford, 81, 00133 Rome, Italy; 3 Unit of Medical Statistics and Molecular Epidemiology, Università Campus Bio-Medico di Roma, Via Álvaro del Portillo 21, 00128 Rome, Italy; f.branda@unicampus.it (F.B.);

**Keywords:** malignant pleural effusion, surgical pleurodesis, systemic immune-inflammation index, neutrophil-to-lymphocyte ratio, biomarkers, unsupervised clustering

## Abstract

**Background**: Pleurodesis is widely used to control malignant pleural effusion (MPE), but reliable pre-procedural predictors of success remain lacking. We assessed whether routinely available systemic inflammatory biomarkers predict short-term pleurodesis outcome and explored multidimensional inflammatory phenotypes. **Methods**: This retrospective single-center cohort included 144 patients who underwent pleurodesis for MPE between January 2021 and August 2025. Pre-procedural systemic immune-inflammation index (SII), neutrophil-to-lymphocyte ratio (NLR), platelet-to-lymphocyte ratio (PLR), and C-reactive protein (CRP) were evaluated using receiver operating characteristic analysis. K-means clustering, principal component analysis, and uniform manifold approximation and projection were used for exploratory phenotyping. **Results**: Complete success occurred in 100 patients (69.4%), failure in 35 (24.3%), and partial response in 9 (6.3%). None of the biomarkers discriminated success from failure (AUC: SII, 0.471; NLR, 0.409; PLR, 0.490; CRP, 0.544). Three exploratory clusters showed crude success rates of 88.9%, 72.3%, and 61.5%, respectively, but substantial overlap and lack of external validation precluded clinical interpretation. **Conclusions**: Single pre-procedural systemic inflammatory biomarkers should not be used alone to select patients for pleurodesis. Prospective multicenter studies integrating systemic markers with pleural biology and mechanical factors are warranted.

## 1. Introduction

Malignant pleural effusion (MPE) is a common manifestation of advanced-stage cancer and is associated with dyspnea, impaired quality of life, recurrent hospitalizations, and limited survival [1,2,3]. Management is primarily palliative and aims to achieve sustained symptom control while minimizing the burden of invasive procedures [4,5]. Treatment options for MPE include chemical pleurodesis and the placement of an indwelling pleural catheter (IPC). The choice of treatment should consider lung expandability, expected survival, performance status (PS), and patient preferences [3,5,6]. Successful pleurodesis requires adequate apposition of the visceral and parietal pleura, together with an effective local inflammatory and fibrotic response. Conversely, pleurodesis failure may result from a non-expandable lung, a high pleural tumor burden, excessive and continuous pleural fluid production, or an inadequate inflammatory response [2,5,7].

Given the frailty of patients with advanced-stage cancer, estimating survival and selecting an appropriate pleural intervention are important. The LENT score combines pleural fluid lactate dehydrogenase, ECOG performance status, neutrophil-to-lymphocyte ratio and tumor type to estimate survival in patients with MPE [8]. Survival prediction should be distinguished from prediction of pleurodesis success.

Systemic inflammatory and immune-related indices derived from routine blood tests, including SII, NLR, PLR and CRP, provide readily available measures of peripheral inflammatory status. Their value for predicting pleurodesis outcome requires evaluation rather than extrapolation from survival models. In PROMISE, candidate biomarkers of pleurodesis response did not validate, whereas clinical and biological variables were used to develop a survival score [9].

The identification of reliable pre-procedural predictors of pleurodesis success could facilitate more targeted patient selection for pleurodesis rather than IPC placement. However, no circulating inflammatory biomarker has been sufficiently validated for the routine prediction of pleurodesis outcomes. Moreover, the relationship between individual inflammatory biomarkers and pleurodesis success may be complex and influenced by the interplay of multiple patient- and disease-related factors [7,9,10]. A multidimensional assessment of inflammatory biomarkers may therefore help identify clinically relevant patient phenotypes and patterns associated with pleurodesis outcomes. Such exploratory approaches, however, require independent validation before they can be incorporated into clinical decision-making.

This study aimed to assess the potential role of pre-procedural systemic inflammatory biomarkers in identifying patients at increased risk of procedural failure and in predicting an unfavorable short-term prognosis.

## 2. Materials and Methods

### 2.1. Study Population and Data Source

This was a single-center retrospective cohort study including all consecutive patients who underwent VATS talc pleurodesis for malignant pleural effusion (MPE) between January 2021 and August 2025 at an Italian academic hospital. Ethical Committee approval was waived due to the retrospective analysis of the study according to national laws regulating observational retrospective studies (Italian law nr. 11960, released on 13 July 2004). Individual patient data were anonymized prior to analysis and stored on a password-protected institutional server with restricted access. No additional patient contact or biological sampling was performed beyond routine clinical practice.

Inclusion criteria were (i) age ≥ 18 years; (ii) pleural fluid with a histologically or cytologically confirmed malignant etiology; (iii) pleurodesis performed with curative or palliative intent; and (iv) availability of at least one post-procedural follow-up assessment. Exclusion criteria were (i) benign pleural effusion without direct pleural involvement; (ii) previous ipsilateral pleurodesis; and (iii) incomplete procedural records.

Clinical, laboratory, and procedural data were extracted from electronic health records and prospectively maintained institutional databases. Variables collected included patient demographics (age, sex, smoking history, body mass index [BMI], and Eastern Cooperative Oncology Group [ECOG] performance status); primary tumor type and histological subtype; molecular mutation profile; previous oncological treatments; disease extent, including the presence, number, and location of distant metastases, as well as lymphangitic carcinomatosis; systemic inflammatory and immune-related biomarkers, including the neutrophil-to-lymphocyte ratio (NLR), platelet-to-lymphocyte ratio (PLR), systemic immune-inflammation index (SII), and C-reactive protein (CRP); pleural effusion quantification by chest radiography; biochemical and cytological pleural fluid analyses; procedural characteristics, including surgical technique and drainage volume; and post-procedural outcomes, including pleurodesis success, complications, time to drain removal, and length of hospital stay.

The primary endpoint was pleurodesis success, defined as complete radiological resolution of the pleural effusion without clinically significant re-accumulation requiring repeat pleural intervention within 30 days of the procedure. Outcome was assessed by chest computed tomography (CT) at 30 days. The CT images were interpreted and formally reported by a radiologist and also reviewed by the treating surgeon. Outcome assessment was not blinded to systemic inflammatory biomarker values. Partial response was defined as residual pleural effusion qualitatively assessed as moderate (non-massive), without a numerical imaging threshold. Pleurodesis outcome was categorized as a three-level variable (success, failure, or partial response) for descriptive and exploratory analyses and as a binary outcome (success vs. failure) for the primary ROC analysis, with partial responders excluded from the latter analysis.

Blood samples used for biomarker assessment were obtained within 30 days before pleurodesis. CRP was measured as part of routine clinical blood testing using the clinical laboratory’s routine CRP assay and expressed in mg/dL. The specific analytical assay method could not be identified from the documentation available for this retrospective study.

Talc was administered exclusively as poudrage during video-assisted thoracoscopic surgery (VATS), with a standard dose of 9 g and a low dose of 7 g. No patients received talc slurry through a chest tube; therefore, a direct VATS-versus-slurry comparison was not possible. The four patients receiving low-dose talc were insufficient for a reliable comparative assessment of doses. In 88 patients (62% of those with available procedural data), a diagnostic pulmonary wedge resection was performed in addition to parietal pleural biopsy and talc pleurodesis to obtain pulmonary tissue for histopathological diagnosis. The recorded “wedge resection + talc” category identifies this additional pulmonary resection; it does not indicate omission of pleural biopsy or coding of pleural biopsy as wedge resection.

The secondary endpoint was overall survival (OS), defined as the time from the date of pleurodesis to death from any cause or the date of the last available follow-up. Patients who were alive at the end of follow-up were treated as censored observations.

### 2.2. Data Preprocessing

All variables were systematically reviewed for consistency and plausibility prior to analysis. Non-numeric entries resulting from data-entry artefacts were coerced to numeric values using a standardized conversion algorithm; irrecoverable entries were treated as missing.

Variables with >50% missingness were excluded from analyses. Missing values in the feature matrix were imputed using median imputation applied independently to each variable. Feature values were standardized to zero mean and unit variance prior to distance-based procedures (clustering).

### 2.3. Statistical Analysis

#### 2.3.1. Descriptive Statistics

Continuous variables are reported as median with interquartile range, as clinical and laboratory variables commonly show skewed distributions. Categorical variables are reported as absolute frequencies and percentages. Group comparisons for continuous variables were performed using the Mann–Whitney U test for two independent groups and the Kruskal–Wallis test for three or more groups. For categorical variables, Pearson’s χ^2^ test was used when assumptions on expected cell counts were met, while Fisher’s exact test was applied in sparse contingency tables. Pairwise associations between continuous variables were assessed using Spearman’s rank correlation coefficient. A two-sided significance threshold of α = 0.05 was adopted.

#### 2.3.2. Predictive Performance of Inflammatory Biomarkers

To evaluate the discriminative ability of systemic inflammatory indices (SII, NLR, PLR, CRP) for pleurodesis outcome, receiver operating characteristic (ROC) curve analysis was performed comparing patients who achieved successful pleurodesis with those in whom the procedure failed, excluding partial responders from the binary analysis. The area under the ROC curve (AUC) was calculated as the primary performance metric. The optimal cut-off value for each biomarker was determined using the Youden index, defined as the point on the ROC curve maximizing sensitivity plus specificity. Sensitivity and specificity at the optimal cut-off were reported. Pleurodesis success was the positive class, and higher biomarker values were treated as indicating a higher probability of success. An AUC below 0.5 therefore denotes an inverse association under this convention. Because the analysis was retrospective and no protocol was registered, this describes the direction used rather than a prospectively pre-specified direction.

Distributions of biomarker levels across outcome categories (Success, Failure, Partial) were visualized using boxplots. Mann–Whitney U tests were used to compare biomarker levels between the Success and Failure groups. Correlations among inflammatory markers and relevant clinical parameters (age, creatinine, lymphocyte percentage, neutrophil percentage) were assessed using Spearman’s rank correlation coefficient and visualized as a heatmap.

Stratified analyses were performed by primary tumor type (Lung, Breast, Pleura) and histology (Adenocarcinoma, Mesothelioma, Ductal, Squamous cell) for subgroups with at least five patients. Success rates were calculated for each tumor type and histology. SII and NLR levels were compared between Success and Failure groups within each tumor type using Mann–Whitney U tests. Median biomarker values (NLR, PLR, SII) for each tumor type stratified by outcome were visualized as a heatmap. ROC curves were additionally generated restricted to lung cancer patients only, representing the largest homogeneous subgroup.

#### 2.3.3. Unsupervised Clustering and Phenotype Identification

K-means clustering was used to identify latent patient phenotypes based on a selected set of clinical and laboratory features including age, ECOG performance status, SII, lymphocyte percentage, neutrophil percentage, pleural effusion quantification (mm), and drainage volume (mL). Features were standardized to zero mean and unit variance prior to clustering to ensure equal contribution of each variable.

The optimal number of clusters was determined using the elbow method applied to the within-cluster sum of squares (inertia) as a function of the number of clusters K. The elbow heuristic, which identifies the point where the marginal decrease in inertia diminishes appreciably, was used to select the final number of clusters.

Uniform Manifold Approximation and Projection (UMAP) was used for nonlinear dimensionality reduction to visualize the clustering structure in two dimensions. Principal component analysis (PCA) was additionally performed as a linear baseline method to quantify global variance structure and to provide an alternative visualization of cluster separation. The proportion of variance explained by the first two principal components was reported.

Cluster profiles were summarized using Z-score standardization of the input features, allowing comparison across heterogeneous biomarkers and clinical variables. Cluster assignments were compared with pleurodesis outcome (Success, Failure, Partial) to assess whether clusters captured prognostic information beyond the binary outcome. Clinical characterization of each cluster included analysis of SII levels, age distribution, pleurodesis success rate, primary tumor composition, and lymphocyte percentage. Between-cluster comparisons for continuous variables were performed using the Kruskal–Wallis test followed by Dunn–Bonferroni post-hoc comparisons where appropriate.

### 2.4. Software and Reproducibility

All analyses were performed using Python version 3.11. Data preprocessing, including median imputation and feature standardization, was performed using pandas and scikit-learn. Descriptive statistical analyses, non-parametric group comparisons, correlation analyses, and ROC curve analyses were performed using SciPy and scikit-learn. K-means clustering and PCA were implemented using scikit-learn, while UMAP was performed using the umap-learn package. Data visualization was performed using standard Python plotting and statistical graphics libraries. ChatGPT (OpenAI; version GPT-6) assisted with wording, organization and presentation of previously reported results during manuscript revision. No new statistical analyses were performed using this tool during revision. The authors remain responsible for checking the revised text and figures against the study records and results.

To ensure reproducibility, a fixed random seed of 42 was used for all stochastic procedures, including clustering and dimensionality-reduction algorithms.

## 3. Results

### 3.1. Study Cohort

A total of 191 patients underwent pleurodesis for malignant pleural effusion. After excluding patients with unknown outcomes or those who died before outcome assessment, 144 patients were included in the final analysis. Baseline clinical and oncological characteristics stratified by pleurodesis outcome are reported in Table 1.

The cohort had a median age of 72 years (IQR 63–79), with no significant difference between patients with successful pleurodesis and those with failure or partial response (*p* = 0.574). Sex distribution was balanced overall (51% male), with a non-significant trend toward a higher proportion of males in the failure/partial group (59% vs. 47%, *p* = 0.248). Smoking status was heterogeneous, with 35% never smokers, 22% current smokers, and 43% former smokers, without differences across outcome groups (*p* = 0.664).

BMI was available for a subset of patients and showed a median value of 24.7 kg/m^2^ (IQR 23.4–27.8), consistent with a predominantly normal-to-overweight population. No significant association with outcome was observed (*p* = 0.211). Functional status, measured using ECOG performance scale, was predominantly favorable, with 57% of patients classified as fully active and 33% as restricted in physically strenuous activity but ambulatory. The distribution of functional status did not differ significantly between groups (*p* = 0.489).

Lung cancer represented the most frequent primary tumor (65%), followed by pleural malignancies (9.0%) and breast cancer (8.3%), with a broad distribution of other tumor types reflecting the heterogeneity of the cohort. No statistically significant differences in primary tumor distribution were observed between outcome groups (*p* = 0.870). Similarly, histological subtypes were heterogeneous, with adenocarcinoma as the predominant type (61%), followed by mesothelioma (8.3%) and ductal carcinoma (9.0%). Histology was not associated with pleurodesis outcome (*p* = 0.854).

The vast majority of patients (94%) had evidence of distant metastatic disease, with no significant difference between groups (*p* = 0.344). Lymphangitic carcinomatosis was present in 62% of patients and similarly distributed across outcomes (*p* = 0.909). At the time of pleurodesis, patients were receiving a range of systemic treatments, most commonly chemotherapy (35%), followed by chemoimmunotherapy (17%) and no active treatment (21%). Although a higher proportion of patients in the failure/partial group received best supportive care (14% vs. 2%), this difference did not reach statistical significance (*p* = 0.233).

Regarding procedural characteristics, the most frequently performed intervention was wedge resection combined with talc pleurodesis (62%), followed by standard talc pleurodesis (33%). Procedure type was not significantly associated with outcome (*p* = 0.594).

Key inflammatory and biochemical indices showed substantial variability across the cohort. The systemic immune-inflammation index (SII) was available in nearly all patients, with a median value of 1284 (IQR 794.7–2343.0), confirming a right-skewed distribution typical of advanced malignancy. The neutrophil-to-lymphocyte ratio (NLR) and platelet-to-lymphocyte ratio (PLR) were available in a more limited subset, with median values of 4.5 (2.7–7.4) and 184.4 (138.9–295.5), respectively. Median creatinine was 0.9 mg/dL (0.7–1.1), while lymphocyte and neutrophil percentages were 15.1% (10.1–23.3) and 72.8% (64.6–80.3), respectively. Radiographic quantification of pleural effusion showed a median size of 70 mm (50–110), and median drainage volume was 1500 mL (1300–2000).

When stratified by outcome, patients with successful pleurodesis consistently exhibited numerically lower inflammatory indices compared with those with failure or partial response, including lower median NLR (3.9 vs. 5.6), PLR (178.0 vs. 188.8), and SII (1241 vs. 1337). These numerical differences were exploratory and not statistically significant (all *p* > 0.05); no conclusion regarding an association between systemic inflammatory burden and pleurodesis efficacy can be drawn.

For descriptive comparisons, failure and partial response were combined as an unsuccessful outcome; partial responders were excluded from the primary binary ROC analyses. Among the 144 evaluable patients, 100 (69%) achieved complete success, defined as full radiological resolution without the need for further intervention. Thirty-five patients (24%) experienced treatment failure, and nine (6.3%) had a partial response; these two groups were combined into a single negative outcome category (*n* = 44, 31%). Follow-up duration tended to be longer among patients with successful pleurodesis (median 11 months vs. 6 months), although this difference approached but did not reach statistical significance (*p* = 0.060).

### 3.2. Biomarker Analysis

#### 3.2.1. Diagnostic Performance of Systemic Inflammatory Indices

To evaluate the potential role of systemic inflammatory indices in predicting pleurodesis outcome, we analyzed four biomarkers including SII, NLR, PLR and CRP. Diagnostic performance was assessed using ROC curve analysis comparing patients who achieved successful pleurodesis (*n* = 96) with those in whom the procedure failed (*n* = 34), excluding partial responders from the binary analysis.

**Figure 1:**
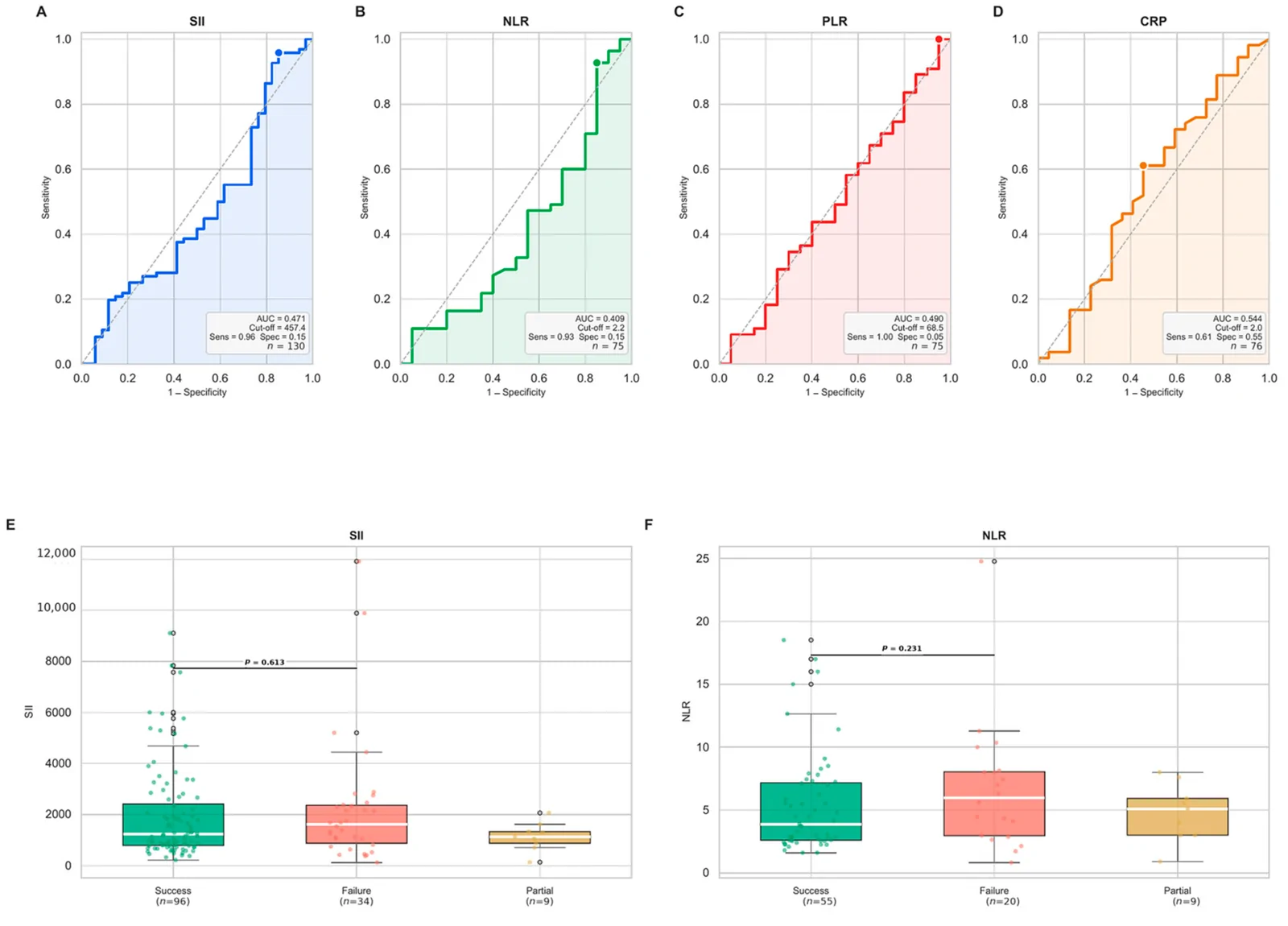

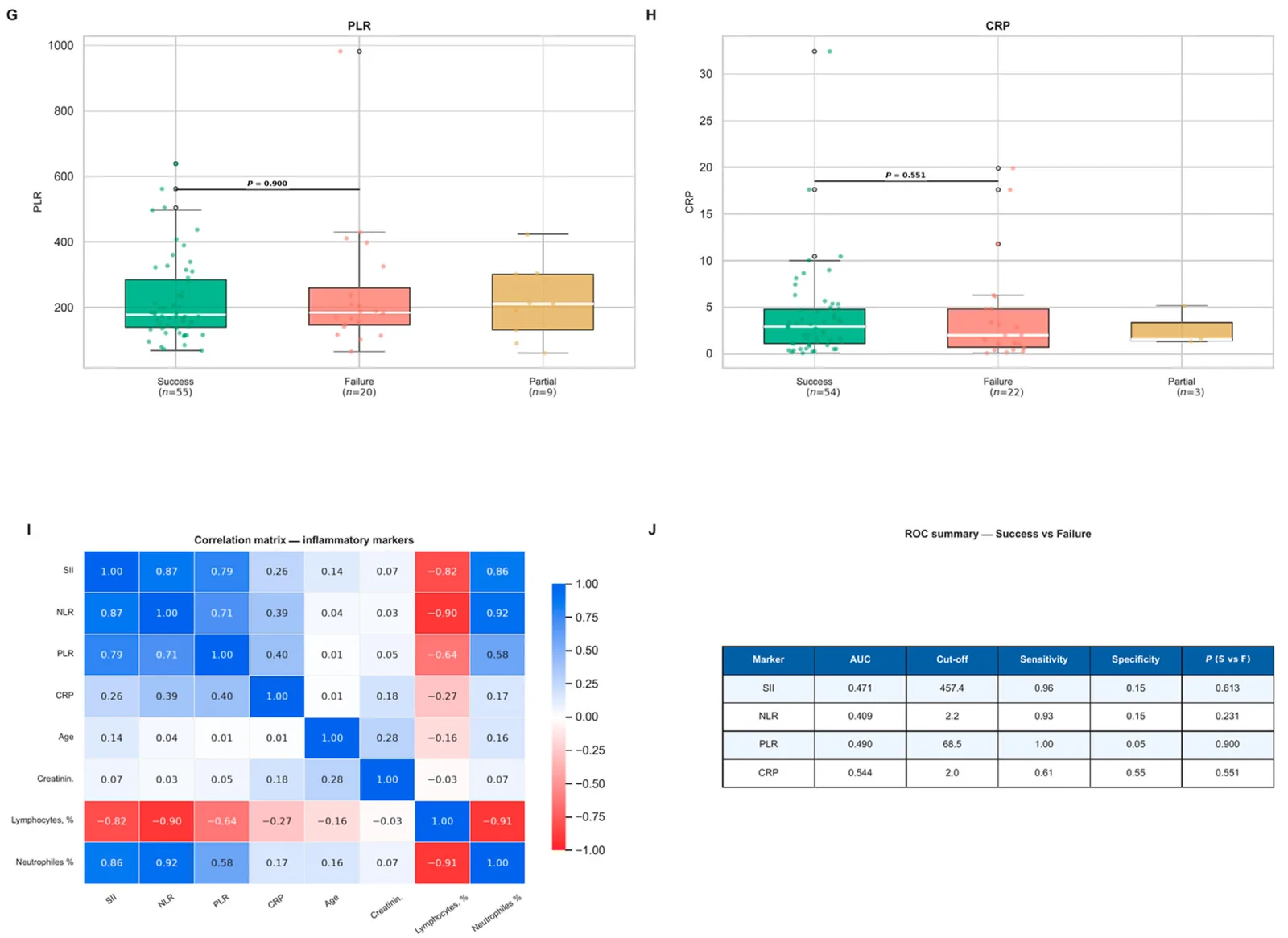
Inflammatory biomarkers (SII, NLR, PLR, CRP) and their association with pleurodesis outcome. (**A**–**D**) ROC curves for successful versus failed pleurodesis, showing AUC, cut-off, sensitivity, specificity, and sample size. (**E**–**H**) Boxplots of biomarker levels across Success, Failure, and Partial groups; *p*-values (Mann–Whitney U test) compare Success versus Failure. (**I**) Spearman correlation matrix among inflammatory markers and clinical parameters (age, creatinine, lymphocyte %, neutrophil %). (**J**) Summary of diagnostic performance metrics including AUC, optimal cut-off, sensitivity, specificity, and *p*-values. Abbreviations: SII, systemic immune-inflammation index; NLR, neutrophil-to-lymphocyte ratio; PLR, platelet-to-lymphocyte ratio; CRP, C-reactive protein; ROC, receiver operating characteristic; AUC, area under the ROC curve. The gray dashed diagonal in ROC panels represents chance-level discrimination. The colored markers on ROC curves denote plotted operating points. In boxplots, the central horizontal line indicates the median, the box the interquartile range, the whiskers variability beyond the box, and individual dots the patient-level observations (including outliers); horizontal comparison bars indicate the pairwise tests. In the correlation heatmap, cell colors and the annotated coefficients represent the direction and magnitude of Spearman correlation; white and dark numerals are used for contrast against the cell background.

Figure 1A displays the ROC curve for SII, which yielded an AUC of 0.471, indicating little discrimination under the direction used. The optimal cut-off value derived from the Youden index was 457.4, which produced a sensitivity of 0.96 but a specificity of only 0.15, meaning that while the test correctly identified 96% of successes, it misclassified 85% of failures as successes. Figure 1B shows the ROC curve for NLR, which yielded an AUC of 0.409; this denotes an inverse association under the specified direction, with lower NLR values tending to accompany success. At the optimal cut-off of 2.2, sensitivity was 0.93 with specificity of 0.15, mirroring the pattern observed for SII. Figure 1C presents the ROC curve for PLR (AUC = 0.490), with a cut-off of 68.5 achieving perfect sensitivity (1.00) at the expense of extremely low specificity (0.05), essentially classifying nearly all patients as potential successes. Figure 1D illustrates the ROC curve for CRP, which yielded an AUC only slightly above 0.5 (AUC = 0.544). At the optimal cut-off of 2.0 mg/dL, sensitivity and specificity were 0.61 and 0.55, respectively, indicating that CRP correctly classified only slightly more than half of the patients.

Mann–Whitney U tests comparing biomarker levels between the Success and Failure groups are shown in Figure 1E–H. For SII (Figure 1E), the difference was not statistically significant (*p* = 0.613). Similarly, NLR (Figure 1F) showed no significant difference (*p* = 0.231), despite slightly higher median values in the Failure group (approximately 4–5) compared with the Success group (approximately 3). PLR (Figure 1G) yielded a *p*-value of 0.900, while CRP (Figure 1H) gave *p* = 0.551, confirming that none of the four biomarkers differed significantly between patients with successful versus failed pleurodesis. Boxplot inspection revealed considerable overlap in biomarker distributions across the three outcome categories (Success, Failure, and Partial) for all markers. SII values extended beyond 10,000 in both success and failure groups, PLR demonstrated wide dispersion from 200 to over 800 across all categories, and CRP levels were notably elevated in both success and failure groups (medians approximately 2–5 mg/dL), with partial responders showing the lowest values (median approximately 1 mg/dL, *n* = 3).

Figure 1I presents the Spearman correlation matrix among inflammatory markers and relevant clinical parameters. Strong positive correlations were observed among the composite indices: SII correlated highly with NLR (ρ = 0.87) and PLR (ρ = 0.79), while NLR and PLR were also strongly correlated (ρ = 0.71), reflecting their shared mathematical dependence on neutrophil, lymphocyte, and platelet counts. All three indices exhibited strong inverse correlations with lymphocyte percentage (ρ = −0.82 to −0.90) and strong positive correlations with neutrophil percentage (ρ = 0.86 to 0.92), consistent with their definitions. CRP demonstrated only weak to moderate correlations with SII, NLR, and PLR (ρ = 0.26–0.40), suggesting that CRP captures distinct aspects of systemic inflammation not fully represented by peripheral blood cell ratios. Age and creatinine showed negligible correlations with all inflammatory markers.

Figure 1J provides a summary of diagnostic performance metrics for all four biomarkers. Under the specified direction, reflected AUCs (1 − AUC) were 0.529 for SII, 0.591 for NLR, 0.510 for PLR, and 0.456 for CRP. The reflected NLR AUC indicates that lower values tended to accompany success, but the group comparison was not statistically significant (*p* = 0.231). None of the biomarkers demonstrated clinically meaningful discrimination, and these findings do not support use of the derived thresholds for clinical decision-making.

#### 3.2.2. Stratification by Tumor Type and Histology

**Figure 2:**
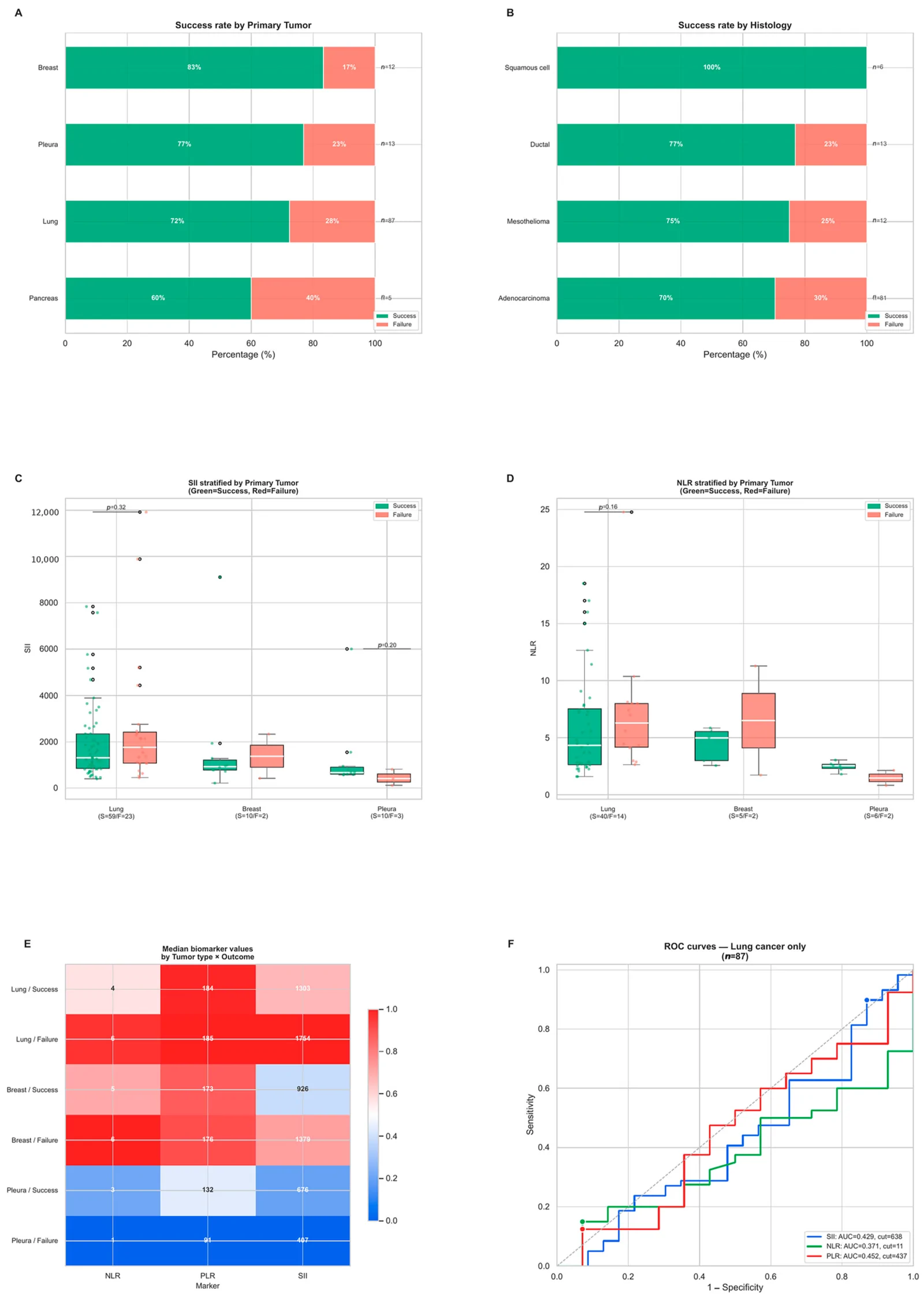
Stratification of inflammatory biomarkers by tumor type and histology. (**A**) Success rate by primary tumor type, showing the percentage of successful pleurodesis (green) versus failure (red) for tumors with at least five patients (Pancreas, Lung, Pleura, Breast). (**B**) Success rate by histology subtype for categories with at least five patients (Adenocarcinoma, Mesothelioma, Ductal, Squamous cell). (**C**) Boxplots of SII levels stratified by tumor type (Lung, Breast, Pleura) and outcome (Success vs. Failure), with Mann–Whitney *p*-values for the Success-Failure comparison within each tumor type. (**D**) Boxplots of NLR levels stratified by tumor type and outcome, with corresponding *p*-values. (**E**) Heatmap displaying median biomarker values (NLR, PLR, SII) for each tumor type (Lung, Breast, Pleura) stratified by outcome (Success vs. Failure). (**F**) ROC curves for SII, NLR, and PLR restricted to lung cancer patients only (*n* = 87), with AUC values and optimal cut-offs. Abbreviations: SII, systemic immune-inflammation index; NLR, neutrophil-to-lymphocyte ratio; PLR, platelet-to-lymphocyte ratio; ROC, receiver operating characteristic; AUC, area under the ROC curve. Dashed diagonals in panel F indicate the no-discrimination reference. In panels C and D, dots are individual observations and boxplots summarize median, interquartile range and variability; horizontal bars identify comparisons with the displayed *p*-values. In panel E, each annotated value is the median on the original biomarker scale, whereas the heatmap color intensity provides a relative visual comparison; white and dark annotations ensure legibility on contrasting backgrounds. Sample sizes are shown next to category labels.

Given the heterogeneity of primary malignancies underlying malignant pleural effusion, we examined whether the association between inflammatory biomarkers and pleurodesis outcome differed according to tumor type and histology. Figure 2 presents stratified analyses for tumors and histology with sufficient sample size (≥5 patients).

Figure 2A illustrates the success rate by primary tumor type. Lung cancer represented the largest subgroup (*n* = 87), with a success rate of 72% (63 patients) versus failure in 28% (24 patients). Pleura (mesothelioma, *n* = 13) showed a success rate of 77% (10 patients), while breast cancer (*n* = 12) achieved 83% success (10 patients). Pancreas cancer, though limited by small sample size (*n* = 5), demonstrated a success rate of 60% (3 patients). Among histologic subtypes (Figure 2B), adenocarcinoma comprised the largest group (*n* = 81), with 70% success (57 patients) and 30% failure (24 patients). Notably, squamous cell carcinoma (*n* = 6) achieved 100% success, while mesothelioma (*n* = 12) and ductal carcinoma (*n* = 13) showed success rates of 75% and 77%, respectively.

Figure 2C displays SII levels stratified by tumor type (Lung, Breast, Pleura) and outcome. In lung cancer patients, SII levels were comparable between the Success (median approximately 1300) and Failure (median approximately 1750) groups, with no statistically significant difference (*p* = 0.32). Similarly, breast cancer patients showed no difference (*p* = 0.20), though the sample size was limited (Success *n* = 10, Failure *n* = 2). Pleura (mesothelioma) patients exhibited numerically lower SII values in both outcome groups, again without significant difference.

Figure 2D presents analogous stratification for NLR. Lung cancer patients showed median NLR values of approximately 4 in the Success group versus 6 in the Failure group, though this difference did not reach statistical significance (*p* = 0.16). Breast and pleura groups demonstrated comparable NLR levels between outcomes, with *p*-values not reported due to small sample sizes.

Figure 2E provides a heatmap of median biomarker values (NLR, PLR, SII) for each tumor type stratified by outcome. Among lung cancer patients, those with failed pleurodesis consistently showed higher median values across all three markers (NLR: 6 vs. 4; PLR: 185 vs. 184; SII: 1754 vs. 1303) compared with successful outcomes. This pattern was less consistent in breast cancer (SII: 1379 vs. 926 favoring success; PLR: 176 vs. 173 essentially equal) and pleura (mesothelioma), where failed patients paradoxically showed lower values across all markers (NLR: 1 vs. 3; PLR: 91 vs. 132; SII: 407 vs. 676).

Figure 2F shows ROC curves restricted to lung cancer patients only (*n* = 87), representing the largest homogeneous subgroup. Discriminative performance for all three markers remained poor: SII achieved an AUC of 0.429 (cut-off 638), NLR yielded an AUC of 0.371 (cut-off 11), and PLR showed an AUC of 0.452 (cut-off 437). These values indicate limited discrimination under the specified direction, as in the overall cohort, confirming that restricting analysis to a single tumor type does not improve the predictive utility of these inflammatory indices. These stratified analyses indicate that while tumor type influences baseline inflammatory marker levels, neither SII, NLR, nor PLR reliably discriminates between successful and failed pleurodesis within specific tumor subtypes. The observed numerical trends, particularly the consistently higher marker levels in lung cancer patients who failed pleurodesis, did not reach statistical significance; these observations are exploratory and do not establish a true association. These findings suggest that systemic inflammatory indices, as measured at a single pre-procedural time point, may have limited utility in predicting pleurodesis outcome regardless of the underlying malignancy.

### 3.3. Cluster Analysis

#### 3.3.1. Identification of Patient Clusters

**Figure 3:**
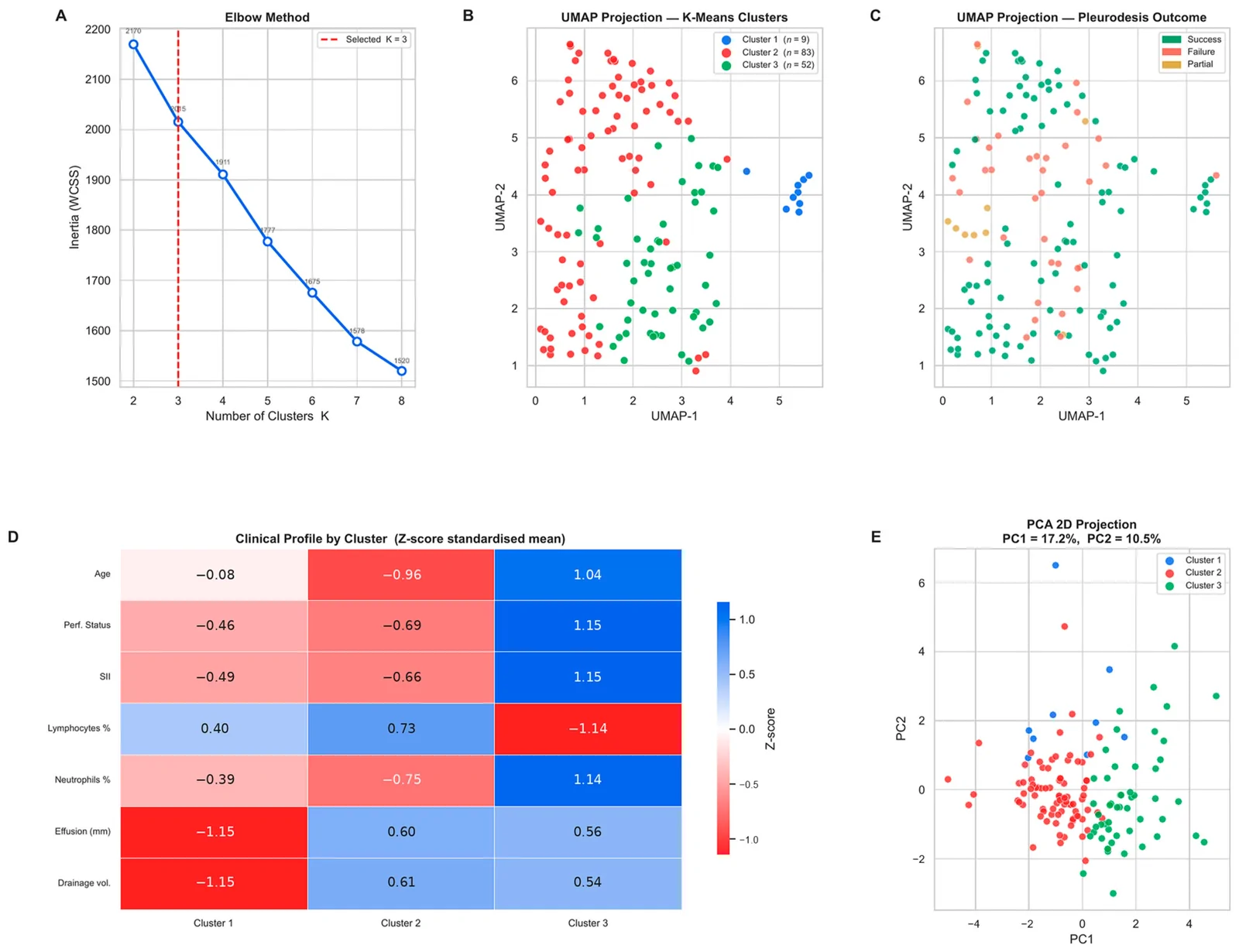
Unsupervised clustering of patients based on clinical and laboratory features. (**A**) Elbow method plot showing within-cluster sum of squares (inertia) as a function of the number of clusters K (range 2–8); the elbow heuristic identifies K = 3 as the optimal number of clusters. (**B**) UMAP two-dimensional projection of all patients (*n* = 144) colored by K-means cluster assignment (Cluster 1, *n* = 9; Cluster 2, *n* = 83; Cluster 3, *n* = 52). (**C**) Same UMAP colored by pleurodesis outcome (Success, Failure, Partial), demonstrating that cluster assignment does not align strictly with outcome. (**D**) Z-score standardized mean values of selected clinical features (Age, Performance Status, SII, Lymphocytes %, Neutrophils %, Pleural effusion quantification in mm, Drainage volume) for each cluster, highlighting distinct clinical profiles. (**E**) PCA two-dimensional projection of patients colored by cluster assignment, with the first two principal components explaining 17.2% and 10.5% of the total variance, respectively. Abbreviations: WCSS, within-cluster sum of squares; K, number of clusters; UMAP, uniform manifold approximation and projection; PCA, principal component analysis; SII, systemic immune-inflammation index. The red dashed vertical line in panel A marks the selected K = 3. In the UMAP and PCA scatterplots, each colored dot represents a patient and overlap reflects proximity in the two-dimensional projection, not duplicated records or incomplete observations. In panel D, blue and red heatmap cells indicate positive and negative standardized values, respectively, and white or dark numbers are chosen for contrast against the cell color.

To identify distinct patient subgroups based on their clinical and laboratory profiles, we performed K-means clustering on a selected set of features including age, performance status, SII, lymphocyte percentage, neutrophil percentage, pleural effusion quantification, and drainage volume. The optimal number of clusters was determined using the elbow method, which plots the within-cluster sum of squares (inertia) as a function of the number of clusters K (Figure 3A). The inertia decreased markedly from K = 2 to K = 3 (from 2015 to 1911) and continued to decrease with higher K values; however, the elbow heuristic identified K = 3 as the optimal choice, balancing model complexity with variance explained.

Based on this selection, patients were assigned to three clusters: Cluster 1 (*n* = 9, 6.3% of the cohort), Cluster 2 (*n* = 83, 57.6%), and Cluster 3 (*n* = 52, 36.1%). UMAP dimensionality reduction was employed to visualize the clustering structure in two dimensions (Figure 3B). The projection revealed that Cluster 2 and Cluster 3 occupy largely overlapping regions of the feature space, while Cluster 1 appears more separated, consistent with its smaller size and potentially distinct clinical profile. When the same UMAP was colored by pleurodesis outcome (Success, Failure, Partial) rather than cluster assignment (Figure 3C), no clear separation was observed, indicating that the clusters capture clinical heterogeneity beyond the simple binary outcome.

Figure 3D displays the standardized mean values (Z-scores) of the input features for each cluster, providing a clinical profile of each subgroup: A positive ECOG performance-status Z-score indicates worse function, whereas a negative score indicates better function.

- Cluster 1 (*n* = 9) had near-average age (Z = −0.08), lower SII (−0.49), better-than-average performance status (−0.46), and the smallest pleural effusion and drainage volume (both −1.15).
- Cluster 2 (*n* = 83) was the largest group and had the youngest age (Z = −0.96), the best performance status (−0.69), the lowest SII (−0.66), and the highest lymphocyte percentage (+0.73).
- Cluster 3 (*n* = 52) had the oldest age (Z = +1.04), the worst performance status (+1.15), the highest SII (+1.15), the lowest lymphocyte percentage (−1.14), and the highest neutrophil percentage (+1.14).

PCA confirmed the clustering structure, with the first two principal components explaining 17.2% and 10.5% of the total variance, respectively (Figure 3E). The projection showed partial separation of Cluster 1 from the other two clusters, while Clusters 2 and 3 showed substantial overlap, consistent with the UMAP visualization.

#### 3.3.2. Clinical Characterization and Outcome by Cluster

**Figure 4:**
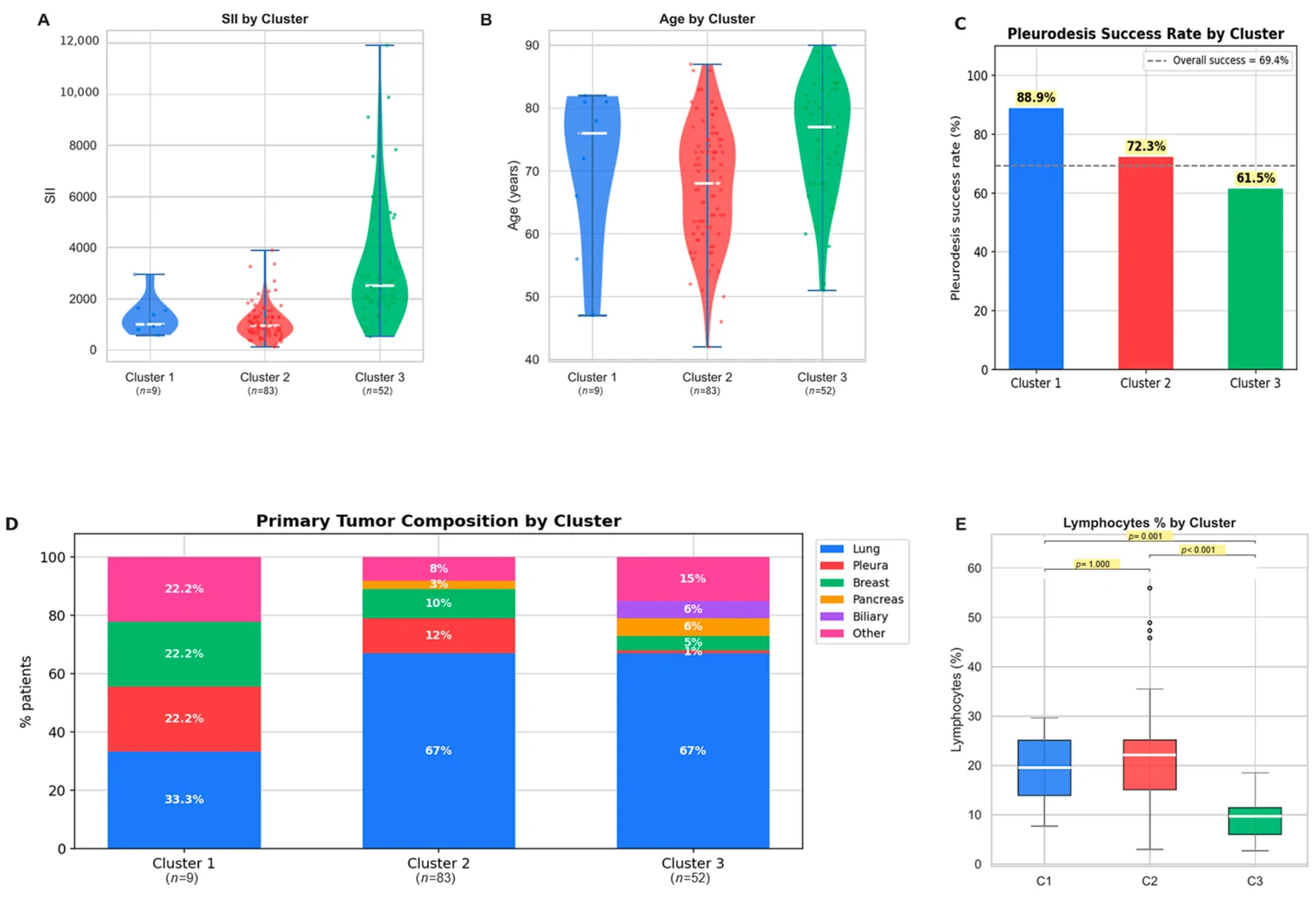
Characterization of the three identified clusters. (**A**) Distribution of SII by cluster, with higher values in Cluster 3. (**B**) Age distribution by cluster; median ages were 77, 76, and 68 years in Clusters 3, 1, and 2, respectively. (**C**) Pleurodesis success rates were 88.9% (8/9), 72.3% (60/83), and 61.5% (32/52) in Clusters 1, 2, and 3; the dashed line indicates overall cohort success (100/144, 69.4%). (**D**) Primary tumor composition by cluster; percentages are descriptive and may not sum to 100% owing to rounding. (**E**) Lymphocyte percentage by cluster. Kruskal–Wallis *p* < 0.001; Dunn–Bonferroni-adjusted comparisons: Cluster 1 versus 2, *p* = 1.000; Cluster 1 versus 3, *p* = 0.001; Cluster 2 versus 3, *p* < 0.001. Median lymphocyte percentages were 19.6%, 22.3%, and 9.7%, respectively. Abbreviations: SII, systemic immune-inflammation index; C1–C3, Clusters 1–3. Dots overlaid on the violin plots in panels A and B represent individual patients, and the white horizontal line indicates the group median; the distribution shape represents the estimated density of values. The gray dashed line in panel C indicates the overall cohort success rate of 69.4%. White and dark percentage labels in panel D were selected to provide contrast against the corresponding colored bar segments. In panel E, the white horizontal bar within each box indicates the median, boxes show the interquartile range, whiskers summarize dispersion, and open circles denote outlying observations. Horizontal comparison brackets indicate adjusted pairwise *p*-values.

Figure 4A presents SII levels across the three clusters. Cluster 3 showed higher SII values, consistent with the Z-score profile in Figure 3D, whereas Clusters 1 and 2 had lower levels. Regarding age distribution (Figure 4B), Cluster 3 was the oldest, with a median age of 77 years, compared with 76 years in Cluster 1 and 68 years in Cluster 2.

The observed pleurodesis success rate varied across clusters (Figure 4C): 89% (8/9) in Cluster 1, 72% (60/83) in Cluster 2, and 62% (32/52) in Cluster 3, compared with 69% in the overall evaluable cohort. These unadjusted descriptive differences were not used to establish predictive or prognostic performance and require confirmation in a larger, independent cohort.

Figure 4D illustrates primary tumor composition within each cluster. Cluster 1 comprised 3 lung cancer cases (33.3%), 2 pleural/mesothelioma cases (22.2%), 2 breast cancer cases (22.2%), and 2 other cases (22.2%). These proportions are purely descriptive given the small cluster size (*n* = 9). Cluster 2 was predominantly composed of lung cancer cases (approximately 67%), with smaller contributions from pleural malignancies (12%), breast cancer (10%), pancreatic cancer (3%), and other tumor types (8%). In Cluster 3, lung cancer remained the most frequent primary tumor (approximately 67%), followed by pancreatic cancer (6%), biliary tract cancer (6%), breast cancer (5%), pleural malignancies (1%), and other types (15%). Biliary tract cancers occurred only in Cluster 3, whereas pancreatic cancers occurred in Clusters 2 and 3. These distributions do not establish a tumor-specific inflammatory phenotype or prognostic association.

Figure 4E shows lymphocyte percentage across clusters. The overall difference was statistically significant (Kruskal–Wallis *p* < 0.001). In Dunn–Bonferroni-adjusted comparisons, Cluster 3 had lower values than Cluster 1 (*p* = 0.001) and Cluster 2 (*p* < 0.001), whereas Clusters 1 and 2 did not differ (*p* = 1.000). Median lymphocyte percentages were 9.7% in Cluster 3, 22.3% in Cluster 2, and 19.6% in Cluster 1. These findings characterize the exploratory clusters and do not establish independent prognostic value.

## 4. Discussion

In this retrospective cohort of patients with malignant pleural effusion, single pre-procedural measurements of systemic inflammatory biomarkers did not provide clinically meaningful discrimination of pleurodesis outcome. SII, NLR, PLR, and CRP showed substantial overlap between patients with successful and unsuccessful pleurodesis, with AUC values close to 0.50. Restricting the analysis to patients with lung cancer, the largest and most homogeneous subgroup, did not improve discriminatory performance. These findings suggest that routinely available peripheral inflammatory indices, when measured at a single pre-procedural time point, are unlikely to be sufficient for identifying patients who will derive greater benefit from pleurodesis. These findings should be interpreted alongside PROMISE, in which candidate biomarkers of pleurodesis response did not validate [9]; the different markers and study designs preclude a direct comparison.

The absence of a clinically useful association between systemic inflammatory indices and pleurodesis success does not imply that inflammation is irrelevant to pleurodesis. Talc-induced pleural injury initiates a local inflammatory cascade involving mesothelial activation, cytokine release, coagulation, and subsequent fibrosis [11,12]. However, the success of pleural symphysis depends on the interaction between this inflammatory response and several local and mechanical determinants, including adequate apposition of the visceral and parietal pleura, lung expandability, pleural tumor burden, pleural fluid characteristics [7]. Systemic indices such as SII, NLR, PLR, and CRP may therefore capture only a limited and nonspecific component of the biological processes underlying pleurodesis. Studies by Zablockis et al., Habal et al. and Ukale et al. evaluated inflammatory changes after pleurodesis, including serum CRP and leukocyte responses [13,14,15]. These postoperative measurements address a different question from the pre-procedural biomarkers assessed here, and reported associations do not establish a validated baseline prediction tool. Their levels may also be influenced by infection, corticosteroid exposure, anticancer treatment, comorbidities, and the timing of blood sampling. This distinction between systemic inflammatory status and local pleural biology may partly explain why peripheral biomarkers showed poor discriminatory performance in our cohort.

Previous studies have investigated local pleural biomarkers and biochemical characteristics as possible predictors of pleurodesis outcome, including sRAGE, pleural fluid glucose and pH [16,17,18]. Their relevance reflects the local pleural environment more directly than peripheral blood indices, but no clinically useful biomarker has yet been sufficiently validated [10]. Pleurodesis success may therefore be better captured by a multidimensional assessment incorporating pleural characteristics, mechanical factors and systemic patient status than by an isolated circulating inflammatory marker [7,10]. Complementary imaging approaches have also been investigated for diagnostic tissue characterization in thoracic oncology, including comparison of conventional endobronchial ultrasound, contrast-enhanced endobronchial ultrasound and elastography [19]. This diagnostic study illustrates complementary characterization in a different thoracic oncology setting; it does not provide evidence of biomarker validity or predict pleurodesis outcomes.

The stratified analyses provide a similar perspective. Among patients with lung cancer, unsuccessful pleurodesis was associated with numerically higher NLR and SII values, but these differences did not reach statistical significance and were not associated with useful discriminatory performance. Moreover, the direction of the observed associations was not consistent across tumor types. These findings should therefore be interpreted as exploratory rather than as evidence of tumor-specific biomarker thresholds. The heterogeneity of malignant pleural effusion, together with the small number of patients in several tumor-specific subgroups, limits the ability to distinguish disease-specific inflammatory effects from random variation.

The unsupervised clustering analysis offers a complementary, hypothesis-generating perspective. Three clinical and inflammatory profiles were identified, with progressively lower observed pleurodesis success across clusters and the least favorable cluster characterized by higher SII, neutrophilia, and lymphopenia. Importantly, pleurodesis outcome was not used to derive the clusters, suggesting that these profiles reflect differences in baseline clinical and inflammatory characteristics rather than simple outcome-based stratification. Nevertheless, the substantial overlap between clusters, the small size of the smallest cluster, the absence of cluster stability analysis, and the lack of external validation preclude any inference regarding predictive performance. The clusters should therefore be regarded as exploratory phenotypes that may help generate hypotheses for future studies rather than as clinically actionable patient classifications.

From a clinical perspective, these findings argue against using individual systemic inflammatory biomarkers as standalone tools for selecting patients for pleurodesis. Patient selection should instead continue to incorporate established determinants of treatment suitability, including lung expandability, performance status, expected survival, disease burden, and patient preferences [3,5,20]. In particular, the present findings do not support using an SII, NLR, PLR, or CRP threshold to favor pleurodesis over an indwelling pleural catheter. Drainage strategy and patient-reported symptom control are also relevant considerations for catheter management [21]. Future predictive models may benefit from integrating systemic inflammatory indices with measures that more directly capture pleural biology and mechanical conditions, including pleural fluid characteristics, lung re-expansion, drainage kinetics, and serial rather than single-point biomarker measurements.

This study has several strengths. It reflects real-world practice in a clinically relevant population with malignant pleural effusion, evaluates inexpensive and widely available biomarkers, and reports their discriminatory performance without selectively emphasizing favorable cut-off values. The combination of conventional statistical analyses with exploratory multidimensional phenotyping also provides a broader assessment of whether systemic inflammation can contribute to pleurodesis outcome prediction.

Several limitations should be considered. First, the retrospective single-center design limits generalizability and introduces the possibility of selection and information bias. Second, substantial missingness affected NLR and PLR and may have reduced the precision of biomarker analyses. Third, the heterogeneity of primary malignancies and the small number of unsuccessful procedures within several subgroups limited statistical power for stratified analyses. Fourth, biomarkers were assessed at a single pre-procedural time point, preventing evaluation of temporal changes in systemic inflammation. Fifth, median imputation may have influenced the structure of the distance-based clustering analysis. Finally, partial responses were excluded from the primary ROC analysis and combined with failures in other analyses, introducing some heterogeneity in outcome definitions across analyses. The 30-day endpoint captures short-term radiological outcome and does not establish durable pleurodesis success or exclude later recurrence. Outcome assessment was not blinded to biomarker values, and partial response was classified qualitatively without a quantitative imaging threshold, introducing potential assessment bias and classification variability. Blood sampling up to 30 days before pleurodesis may not fully reflect inflammatory status at the time of the procedure. The specific CRP analytical method was not recoverable from the documentation available for this retrospective analysis, limiting assay reproducibility.

Overall, our findings suggest that systemic inflammatory biomarkers measured before pleurodesis do not provide sufficient discriminatory information to predict procedural success in patients with malignant pleural effusion. Rather than indicating that inflammation has no role in pleurodesis, these results support the hypothesis that treatment response reflects a complex interaction between systemic inflammatory status, local pleural biology, tumor burden, and mechanical factors. Prospective multicenter studies integrating these domains, together with serial biomarker measurements and patient-reported outcomes, are warranted to determine whether multidimensional models can improve individualized selection of pleural interventions.

## 5. Conclusions

The lack of discriminatory value of individual systemic inflammatory biomarkers suggests that pleurodesis outcomes are unlikely to be adequately predicted by isolated peripheral inflammatory measurements and may instead reflect the complex interplay of systemic inflammation, local pleural biology, tumor burden, and mechanical factors such as lung expandability.

## Figures and Tables

**Table 1:** Baseline clinical and oncological characteristics stratified by pleurodesis outcome. Continuous variables: median (IQR). Categorical variables: *n* (%). *p*-values: Wilcoxon rank-sum (continuous), Pearson χ^2^ (categorical). All talc administration was performed during VATS. Diagnostic wedge resection was performed in addition to parietal pleural biopsy. Failure and partial response are combined for the descriptive comparisons in this table; partial responders were excluded from the primary ROC analyses. Abbreviations: BMI, body mass index; ECOG, Eastern Cooperative Oncology Group; SII, systemic immune-inflammation index; NLR, neutrophil-to-lymphocyte ratio; PLR, platelet-to-lymphocyte ratio; IQR, interquartile range; VATS, video-assisted thoracoscopic surgery.

Variable	Overall (*n* = 144)	Success (*n* = 100)	Failure/Partial (*n* = 44)	*p*-Value
**Age (years)**	72.0 (63.0–79.0)	72.0 (62.5–79.0)	72.5 (65.5–79.0)	0.574
**Sex**				0.248
* Male*	73 (51%)	47 (47%)	26 (59%)	
* Female*	71 (49%)	53 (53%)	18 (41%)	
**Smoking status**				0.664
* Never*	50 (35%)	37 (37%)	13 (30%)	
* Current*	32 (22%)	22 (22%)	10 (23%)	
* Former*	62 (43%)	41 (41%)	21 (48%)	
**BMI (kg/m^2^)**	24.7 (23.4–27.8)	25.9 (23.4–27.8)	24.2 (22.9–28.1)	0.211
*Missing*	66	49	17	
**ECOG Performance Status**				0.489
* PS 0*	82 (57%)	59 (60%)	23 (52%)	
* PS 1*	47 (33%)	30 (30%)	17 (39%)	
* PS 2*	11 (7.7%)	7 (7.1%)	4 (9.1%)	
* PS 3*	3 (2.1%)	3 (3.0%)	0 (0%)	
*Missing*	1	1	0	
**Primary tumor Site**				0.870
* Lung*	94 (65%)	63 (63%)	31 (70%)	
* Pleura*	13 (9.0%)	10 (10%)	3 (6.8%)	
* Breast*	12 (8.3%)	10 (10%)	2 (4.5%)	
* Kidney*	2 (1.4%)	1 (1.0%)	1 (2.3%)	
* Lymphoma*	3 (2.1%)	2 (2.0%)	1 (2.3%)	
* Prostate*	2 (1.4%)	2 (2.0%)	0 (0%)	
* Pancreas*	5 (3.5%)	3 (3.0%)	2 (4.5%)	
* Skin*	1 (0.7%)	0 (0%)	1 (2.3%)	
* Ovarian*	0 (0%)	0 (0%)	0 (0%)	
* Sarcoma*	2 (1.4%)	1 (1.0%)	1 (2.3%)	
* Bone*	2 (1.4%)	1 (1.0%)	1 (2.3%)	
* Biliary*	3 (2.1%)	2 (2.0%)	1 (2.3%)	
* Gastric*	1 (0.7%)	1 (1.0%)	0 (0%)	
* Uterus*	0 (0%)	0 (0%)	0 (0%)	
* Thymus*	2 (1.4%)	2 (2.0%)	0 (0%)	
* Larynx*	1 (0.7%)	1 (1.0%)	0 (0%)	
* Other*	1 (0.7%)	1 (1.0%)	0 (0%)	
**Histological subtype**				0.854
* Adenocarcinoma*	88 (61%)	57 (57%)	31 (70%)	
* Mesothelioma*	12 (8.3%)	9 (9.0%)	3 (6.8%)	
* Squamous cell carcinoma*	6 (4.2%)	6 (6.0%)	0 (0%)	
* Ductal carcinoma*	13 (9.0%)	10 (10%)	3 (6.8%)	
* Sarcoma*	4 (2.8%)	3 (3.0%)	1 (2.3%)	
* Clear cell carcinoma*	1 (0.7%)	1 (1.0%)	0 (0%)	
* NOS*	3 (2.1%)	2 (2.0%)	1 (2.3%)	
* Undifferentiated carcinoma*	1 (0.7%)	1 (1.0%)	0 (0%)	
* Lymphoma*	3 (2.1%)	2 (2.0%)	1 (2.3%)	
* Neuroendocrine tumor*	3 (2.1%)	2 (2.0%)	1 (2.3%)	
* Melanoma*	1 (0.7%)	0 (0%)	1 (2.3%)	
* Small cell lung cancer (SCLC)*	3 (2.1%)	2 (2.0%)	1 (2.3%)	
* Cholangiocarcinoma*	2 (1.4%)	1 (1.0%)	1 (2.3%)	
* Epithelioid mesothelioma*	1 (0.7%)	1 (1.0%)	0 (0%)	
* Thymoma*	1 (0.7%)	1 (1.0%)	0 (0%)	
* Other*	2 (1.4%)	2 (2.0%)	0 (0%)	
**Distant metastases**				0.344
* Yes*	134 (94%)	91 (92%)	43 (98%)	
* No*	9 (6.3%)	8 (8.1%)	1 (2.3%)	
* Unknown*	0 (0%)	0 (0%)	0 (0%)	
* Missing*	1	1	0	
**Lymphangitis carcinomatosis, *n* (%)**				0.909
* 1*	55 (38%)	39 (39%)	16 (36%)	
* 2*	89 (62%)	61 (61%)	28 (64%)	
**Systemic therapy**				0.233
* Chemotherapy*	51 (35%)	36 (36%)	15 (34%)	
* Chemoimmunotherapy*	25 (17%)	18 (18%)	7 (16%)	
* Immunotherapy*	7 (4.9%)	4 (4.0%)	3 (6.8%)	
* Radiotherapy*	7 (4.9%)	6 (6.0%)	1 (2.3%)	
* Chemoradiotherapy*	10 (6.9%)	7 (7.0%)	3 (6.8%)	
* Targeted therapy*	3 (2.1%)	3 (3.0%)	0 (0%)	
* Best supportive care*	8 (5.6%)	2 (2.0%)	6 (14%)	
* None*	30 (21%)	22 (22%)	8 (18%)	
* Unknown*	3 (2.1%)	2 (2.0%)	1 (2.3%)	
**Procedure category**				
				0.594
* Talc pleurodesis (standard, 9 g)*	47 (33%)	33 (33%)	14 (33%)	
* Talc pleurodesis (low dose, 7 g)*	4 (2.8%)	3 (3.0%)	1 (2.3%)	
* Diagnostic wedge resection + talc*	88 (62%)	60 (60%)	28 (65%)	
* Other*	4 (2.8%)	4 (4.0%)	0 (0%)	
* Missing*	1	0	1	
**SII**	1284.0 (794.7–2343.0)	1241.0 (787.8–2469.5)	1337.3 (816.0–2291.0)	0.613
* Missing*	5	4	1	
**NLR**	4.5 (2.7–7.4)	3.9 (2.6–7.2)	5.6 (3.0–8.0)	0.231
* Missing*	60	45	15	
**PLR**	184.4 (138.9–295.5)	178.0 (136.0–290.0)	188.8 (141.7–301.0)	0.900
* Missing*	60	45	15	
**Creatinine (mg/dL)**	0.9 (0.7–1.1)	0.9 (0.8–1.1)	0.9 (0.7–1.2)	0.433
* Missing*	4	3	1	
**Lymphocytes (%)**	15.1 (10.1–23.3)	15.7 (10.3–23.9)	13.4 (9.8–22.2)	0.287
* Missing*	5	4	1	
**Neutrophils (%)**	72.8 (64.6–80.3)	72.1 (63.9–80.6)	73.9 (66.5–80.3)	0.640
* Missing*	5	4	1	
**Pleural effusion size (mm)**	70.0 (50.0–110.0)	70.0 (55.0–100.0)	95.0 (50.0–145.0)	0.450
* Missing*	60	44	16	
**Drainage volume (mL)**	1500.0 (1300.0–2000.0)	1500.0 (1500.0–2000.0)	1500.0 (1000.0–2000.0)	0.367
* Missing*	69	47	22	
**Pleurodesis outcome**				
* Success*	100 (69%)	100 (100%)	0 (0%)	
* Failure*	35 (24%)	0 (0%)	35 (80%)	
* Partial*	9 (6.3%)	0 (0%)	9 (20%)	
* Death*	0 (0%)	0 (0%)	0 (0%)	
* Unknown*	0 (0%)	0 (0%)	0 (0%)	
**Follow-up (months)**	10.0 (3.0–24.0)	11.0 (4.0–24.0)	6.0 (2.0–16.0)	0.060
* Missing*	23	12	11	

## Data Availability

The data presented in this study are available on request from the corresponding author due to privacy.

## Abbreviations

AUC	area under the receiver operating characteristic curve
BMI	body mass index
CRP	C-reactive protein
ECOG	Eastern Cooperative Oncology Group
IQR	interquartile range
MPE	malignant pleural effusion
NLR	neutrophil-to-lymphocyte ratio
OS	overall survival
PCA	principal component analysis
PLR	platelet-to-lymphocyte ratio
SII	systemic immune-inflammation index
UMAP	uniform manifold approximation and projection
WCSS	within-cluster sum of squares.
